# Numerical Assessment of Novel Helical/Spiral Grafts with Improved Hemodynamics for Distal Graft Anastomoses

**DOI:** 10.1371/journal.pone.0165892

**Published:** 2016-11-18

**Authors:** Foad Kabinejadian, Michael McElroy, Andres Ruiz-Soler, Hwa Liang Leo, Mark A. Slevin, Lina Badimon, Amir Keshmiri

**Affiliations:** 1 Department of Biomedical Engineering, University of Michigan, 1107 Carl A. Gerstacker Building, 2200 Bonisteel Blvd, Ann Arbor, Michigan, 48109, United States of America; 2 Engineering and Materials Research Centre, Manchester Metropolitan University, Manchester, M1 5GD, United Kingdom; 3 Department of Biomedical Engineering, National University of Singapore, 4 Engineering Drive 3, Block E4 #04–08, Singapore, 117583, Singapore; 4 Healthcare Science Research Centre, Manchester Metropolitan University, Manchester, M1 5GD, United Kingdom; 5 Cardiovascular Research Center, CSIC-ICCC, Hospital de la Santa Creu i Sant Pau (UAB), C/Sant Antoni Maria Claret 167, 08025, Barcelona, Spain; 6 School of Mechanical, Aerospace and Civil Engineering, University of Manchester, M13 9PL, United Kingdom; Technion Israel Institute of Technology, ISRAEL

## Abstract

In the present work, numerical simulations were conducted for a typical end-to-side distal graft anastomosis to assess the effects of inducing secondary flow, which is believed to remove unfavourable flow environment. Simulations were carried out for four models, generated based on two main features of 'out-of-plane helicity' and 'spiral ridge' in the grafts as well as their combination. Following a qualitative comparison against *in vitro* data, various mean flow and hemodynamic parameters were compared and the results showed that helicity is significantly more effective in inducing swirling flow in comparison to a spiral ridge, while their combination could be even more effective. In addition, the induced swirling flow was generally found to be increasing the wall shear stress and reducing the flow stagnation and particle residence time within the anastomotic region and the host artery, which may be beneficial to the graft longevity and patency rates. Finally, a parametric study on the spiral ridge geometrical features was conducted, which showed that the ridge height and the number of spiral ridges have significant effects on inducing swirling flow, and revealed the potential of improving the efficiency of such designs.

## 1 Introduction

### 1.1 Graft Anastomoses

An anastomosis is a surgical connection between an autologous or prosthetic graft and veins or arteries inside the human body. From a geometrical point of view, the graft anastomoses can be divided into three classes: (1) end-to-side, (2) end-to-end, and (3) side-to-side anastomosis.

In general, vascular grafts can be categorised under the following two main applications:

1. **Arterial Bypass Grafts (ABGs):** Examples of ABGs include Peripheral Vascular Disease (PVD) and Coronary Artery Disease (CAD).
2. **Arterio-Venous Grafts (AVGs):** AVGs are mainly used for creating an ‘access point’ for haemodialysis treatments for patients with renal diseases.

### 1.2 Graft Failure

Graft failure is currently a major concern for medical practitioners in treating PVD and CAD. For instance, almost 35,000 Coronary Artery Bypass Graft (CABG) procedures are performed each year in the UK according to the British Heart Foundation; however, over 50% of CABGs fail within 10 years. In 1999, an estimated 688,000 bypass surgeries were performed in the United States but up to 10% of these procedures failed within 30 days of surgery [1]. Similarly, stenosis at the graft-vein junction caused by Intimal Hyperplasia (IH) is the major cause of failure of arterio-venous access grafts used for hemodialysis.

Early graft failure (within 30 days) is attributable to surgical technical errors and resulting thrombosis, while late graft failures are mainly caused by progression of atherosclerosis and IH [2,3]. It is now widely accepted that hemodynamic factors play an important role in the formation and development of IH [4,5]. This point will be discussed further in Section 3.4.

### 1.3 Spiral/Helical Flow

One of the most significant contributions towards improving the hemodynamics forces in distal graft anastomoses was based on a research which showed that the ‘spiral flow’ is a natural phenomenon in the whole arterial system [6,7]. The spiral flow in arteries is caused by the rotational compressive pumping of the heart [8] and is supported by the tapered, curved and non-planar geometry of the arterial system [9,10].

Amongst different designs which have emerged in the past few decades to mimic the physiological blood flow in arteries and grafts, the present study builds up on the following two successful and innovative designs:

1. **‘SwirlGraft’** developed by Caro and colleagues [11] at Veryan Medical Ltd is a new AV shunt graft with a helical out-of-plane geometric feature incorporating ‘Small Amplitude Helical Technology’ (SMAHT). Compared to a conventional ePTFE graft, the animal experiments reported in [11] demonstrated that there was less thrombosis in SwirlGraft. The difference became even more remarkable after 8 weeks of implementation. To date, a number of researchers have simulated out-of-plane grafts geometries that induce 3D swirling [12–17]. Whilst the majority of the previous numerical simulations have studied the helical flow using various mean flow and wall shear stress-based hemodynamics parameters, Cookson et al. [14] used high-order particle tracking, and an information entropy measure to understand and quantify the mixing effect in helical geometries and found the optimal helical geometry, in terms of mixing versus pressure loss. SMAHT has inspired other novel flow field augmentation techniques too including the work of Cookson et al. [18] which has shown that joining together two helical geometries, of different helical radii, would enhance mixing, with minor increase in pressure loss [14].
2. **‘Spiral Flow Graft’** was introduced by Vascular Flow Technologies (VFT) Ltd and consists of a prosthetic ePTFE graft design that is engineered to induce spiral flow through an internal ridge within its distal end. This design is primarily based on research carried out by Stonebridge and colleagues at Ninewells Hospital in Dundee, Scotland on the naturally occurring helical flow found in studies of the cardiovascular system using Doppler ultrasound. The results of an early clinical non-randomised study for VFT peripheral bypass graft were promising and showed primary patency rates of 81% for above-the-knee bypasses and 57.3% for below-the-knee bypasses at 30 months of follow-up. The respective secondary patency rates were 81% and 64% [19].

Interested readers are referred to a recent review by Liu et al. [20] for an extensive overview of the potential clinical applications of helical flows.

### 1.4 Aims and Objectives

While the physiological importance of secondary motion in circulation has clearly been highlighted in the literature [6,7], the benefits of helical/spiral prostheses in vascular conduits have yet to be firmly established [20–22]. The overall aim of the present work is to investigate the hemodynamic effects of inducing secondary flow in a typical end-to-side distal graft anastomosis and to assess its effectiveness in improving the distribution of hemodynamic factors. In order to achieve this aim, Computational Fluid Dynamics (CFD) has been used to simulate four different end-to-side bypass graft models which have been designed based on two vascular flow prostheses, namely ‘SwirlGraft’ and ‘Spiral Flow Graft’ and their combination.

## 2 Methods

### 2.1 Geometric Models

**Fig 1:**
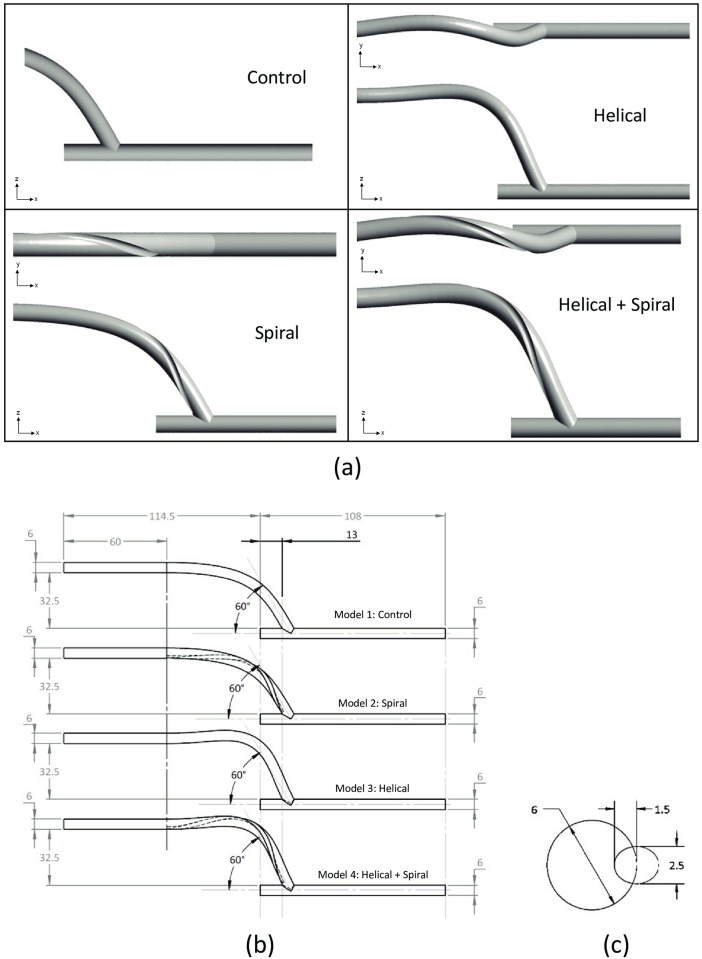
(a) Schematics of the four geometric models used in the present study; (b) Dimensions of the four geometric models used in the present study; (c) Cross-sectional view of the ridge profile used in the ‘Spiral’ and ‘Helical+Spiral’ models (the dimensions shown are of the ellipse that was used to create the ridge. The centre of the ellipse was located at the surface of the graft wall and kept perpendicular to the axis of the graft). All dimensions are in mm.

The main geometry studied in this work represents an End-To-Side (ETS) distal graft anastomosis in a Peripheral Artery Bypass Graft configuration. The dimensions and the schematics of the models studied here are given in Table 1 and Fig 1, respectively. A brief description of each model is as follows:

**Table 1:** Key dimensions and features of the four geometric models studied here.

	Feature		Model 1: Control	Model 2: Spiral	Model 3: Helical	Model 4: Helical+Spiral
**Geometry**	Graft inner diameter (D)		6mm			
	Spiral Pitch		–	82.53mm (13.755D)	–	89.26mm (14.88D)
	Number of spiral turns		–	1	–	1
	Spiral ridge	Height	–	1.5mm	–	1.5mm
		Width	–	2.5mm	–	2.5mm
	Orientation of spiral ridge trailing edge		–	Above heel (6 O’clock)	–	Above heel (6 O’clock)
	Helical amplitude		–		±3mm (0.5D)	
	Length of curved section at the axis		88.53mm (14.755D)		95.19mm (15.87D)	
	Angle of anastomosis		60°			
**Mesh**	Number of cells		1,770,931	2,627,594	1,820,741	2,748,102

**Model 1 (Control Graft):** This model represents a baseline and a conventional ETS distal graft anastomosis for a peripheral artery bypass configuration.

**Model 2 (Spiral Graft):** This model has a one-pitch-long internal spiral inducer within the distal end of the graft in the form of an internal ridge. The geometrical dimensions of the spiral ridge in this model are based on the ‘Spiral Flow Graft’ developed by VFT.

**Model 3 (Helical Graft):** The distal end of the bypass graft in this model consists of a small amplitude out-of-plane helical configuration. The helical section of this model involves a one-turn helix with pitch and amplitude approximately 14*D* and 0.5*D*, respectively (where *D* is the internal diameter of the graft). The pitch of the external helicity in this model is equal to that of the spiral ridge in Model 2, while its amplitude is based on SMAHT design proposed by Caro et al. [11].

**Model 4 (Helical+Spiral Graft):** The distal end of the bypass graft in this model combines the geometrical features present in both Models 2 and 3. Note that in this model the out-of-plane helical and the internal ridge have the same pitch and turn in the same direction.

### 2.2 Computational Domain and Mesh

**Fig 2:**
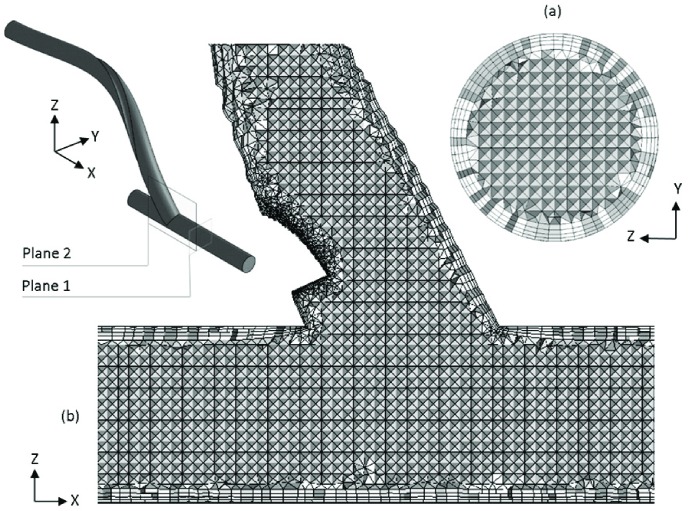
Images of the finite volume mesh used in the present study. (a) Cross-sectional view of the host artery distal of the anastomosis (taken at Plane 1); (b) Cut-away of mesh at the anastomosis (taken at Plane 2). Note that while this image corresponds to Model 4, the mesh for the other 3 models have very similar resolution.

The computational domains used here is based on finite-volume hybrid mesh consisting of prismatic elements for the near-wall and tetrahedral elements for the core regions and were generated using ANSYS-Meshing (Version 14.5). A series of rigorous mesh independency tests were conducted on Model 4, involving over 14 levels of refinement, ranging from 0.5–13.0 million elements. Subsequently, the computational domains with approx. 2.75 million elements were considered to be sufficient here, as further mesh refinement could only result in less than 1% change in velocity and Wall Shear Stress (WSS) at some examined sections. Segments of the volume mesh corresponding to Model 4 are shown in Fig 2.

### 2.3 Governing Equations and Boundary Conditions

The present bypass configuration is assumed to be that of Superficial Femoral Artery (SFA) bypass graft and the blood flow is defined as a three-dimensional, time-dependent, incompressible, isothermal, non-Newtonian and laminar flow [23], whose governing equations are:

Continuity equation,

∇⋅u=0 (1)

and Navier-Stokes equation,

$$\rho \frac{\partial \;u}{\partial \;t}+\rho \left(u\cdot \nabla \right)\;u=-\nabla p+\eta \nabla^{2}u$$ (2)

where ***u*** is the velocity vector, *t* is the time, *p* is the pressure, and *η* is the dynamic viscosity of blood. The density (*ρ*) of blood is assumed to be 1050 kg/m^3^ [24,25], and the Carreau-Yasuda model [26] is employed to model the shear thinning behaviour of blood [27].

The proximal section of the graft is not considered since a number of longer curved graft inlets have been tested for the control model, and it was found that the difference in the secondary flow and vortical structures in the anastomosis was minimal.

**Fig 3:**
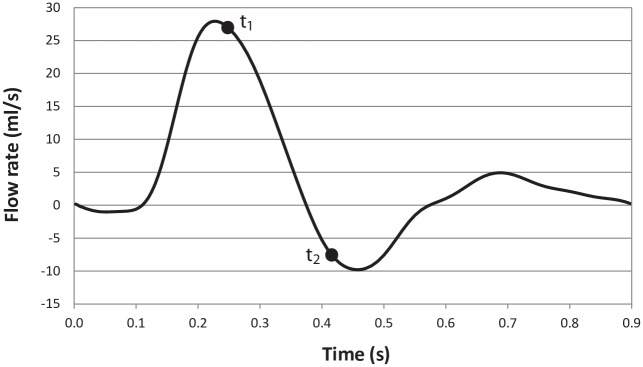
The waveform of the blood flow through the Superficial Femoral Artery (SFA) graft.

A fully-developed pulsatile flow is applied at the graft inlet. The inlet flow waveform, shown in Fig 3, is based on Magnetic Resonance Imaging (MRI) measurements of blood volume flow in femoral artery of a healthy subject [28] with the time period of *T* = 0.9 s. The peak flow rate is *Q*_max_ = 27.9ml/s, and the maximum Reynolds and Womersley numbers are calculated to be *Re*_max_ = 1776 and *α* = 4.34, respectively, based on the conventional Newtonian blood viscosity value of 0.0035 Pa∙s. At the artery outlet, the traction-free outflow boundary condition is applied. No-slip boundary condition is applied to all walls and a rigid wall model is assumed [29,30].

### 2.4 Solver Settings

The governing equations were solved numerically by a finite-volume method and the CFD code, ANSYS-CFX (Version 14.0), using a fully implicit second-order backward Euler differencing scheme. The convergence criterion (a normalised residual, obtained based on the imbalance in the linearized system of discrete equations) was set to 10^−6^ in this study. The time-step size was taken to be 0.01s, and the results were recorded at the end of each time-step. In order to eliminate the start-up effects of transient flow, the computation was carried out for four periods, and the fourth period results are presented.

## 3 Results

### 3.1 Qualitative Comparison against Experimental Data

**Fig 4:**
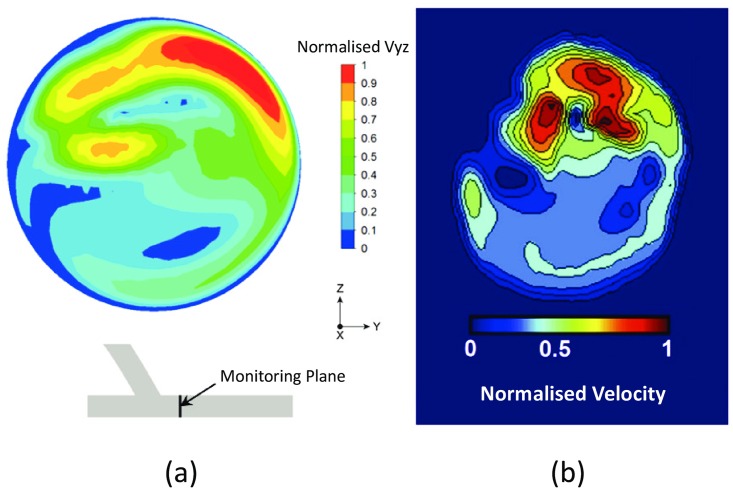
Qualitative comparison of the normalised secondary velocity magnitude against the experimental data of [31]: (a) the present numerical simulations; (b) the experimental data. The results correspond to a plane positioned 5 mm distal from the toe of the anastomosis and *Re* = 1140.

In this section, a series of steady-state simulations were carried out for a spiral-inducing graft configuration, *similar* to the geometry tested experimentally by Kokkalis et al. [31] (note that the present authors have reconstructed the design used in [31] as the exact geometrical data could not be provided by Kokkalis et al. due to commercial sensitivity reasons). In their experiments, the secondary flow motions induced by the Spiral Laminar Flow PV graft (VFT Ltd., Dundee, UK) were compared with those of a control device. Constant flow rates were applied and data were collected in the cross-sectional view distal from the graft outflow and dual-beam vector Doppler was used to create 2-D velocity maps. Fig 4 shows one of the qualitative comparisons for the cross-sectional velocity magnitude at a plane positioned 5 mm distal from the toe of the anastomosis at *Re* = 1140. Relatively good qualitative agreement with the data can be seen, especially in identifying the spiral flow in the host artery and areas with the maximum velocity magnitude. It is important, however, to sound a note of caution in relation to treating the qualitative comparison presented here as ‘validation’. Accuracy of the numerical simulation in problems similar to the one studied here should only be assessed through quantitative comparison against *in vitro* data.

### 3.2 Axial and Secondary Velocity Magnitude

**Fig 5:**
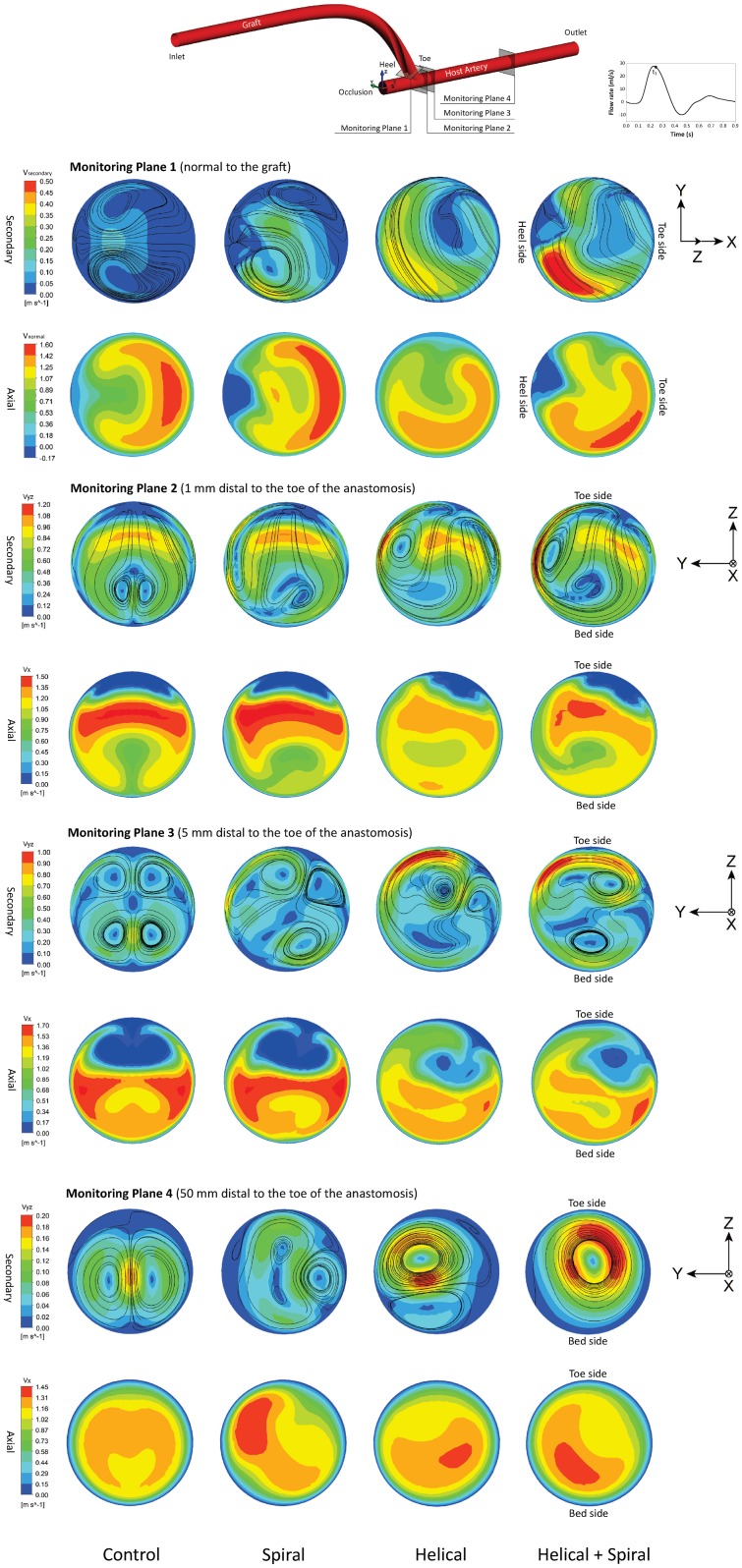
Comparison of the secondary and axial velocity magnitude at approximately peak flow phase (*t*_1_ = 0.25s) at four monitoring planes for all four models.

**Fig 6:**
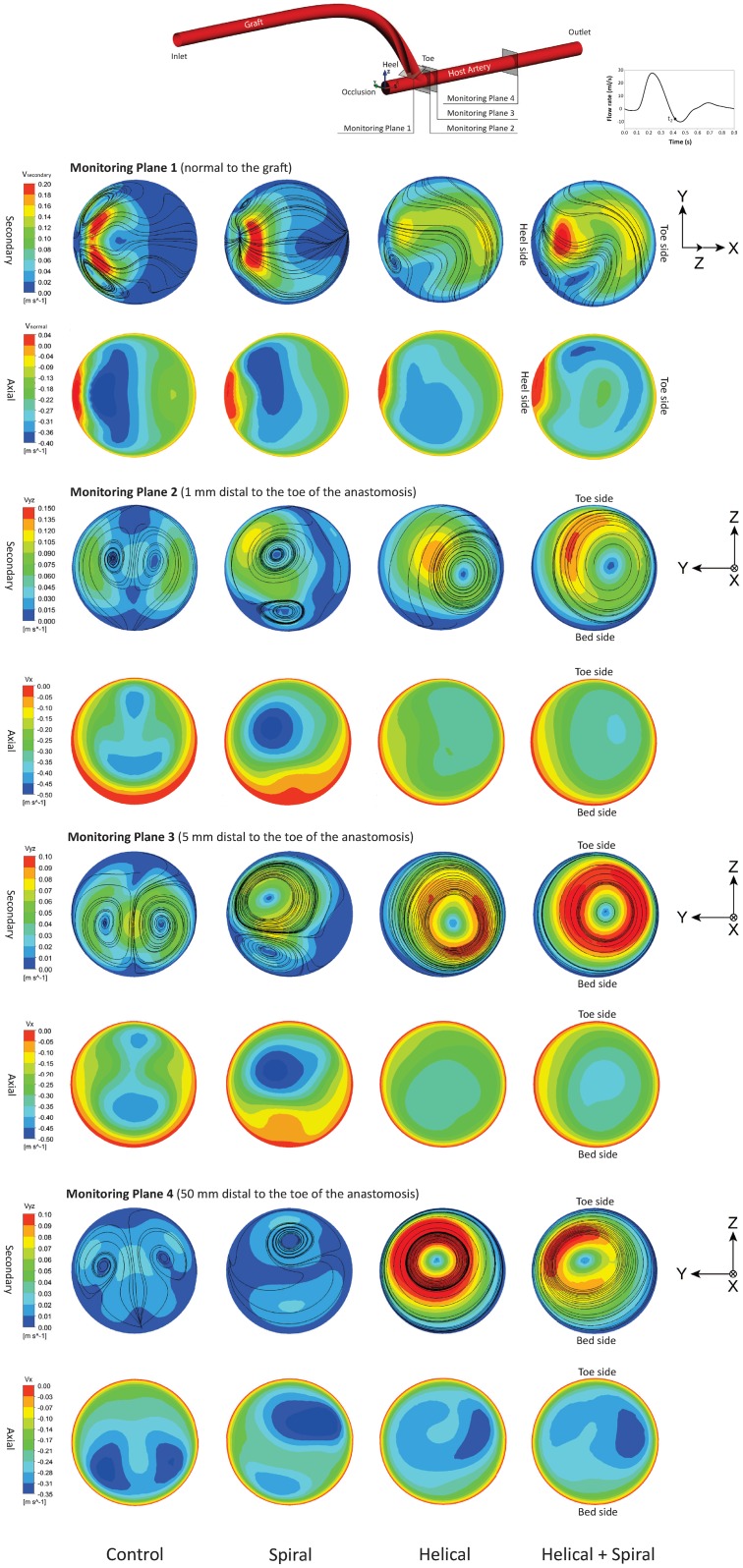
Comparison of the secondary and axial velocity magnitude at approximately reversed flow phase (*t*_2_ = 0.41s) at four monitoring planes for all four models.

Figs 5 and 6 show the axial and secondary velocity magnitude distributions under pulsatile flow condition at the peak flow (*t*_1_ = 0.25s) and reversed flow (*t*_2_ = 0.41s) phases, respectively. The results for all the four models are shown at four different monitoring planes including one normal to the graft at the anastomosis (i.e., Monitoring Plane 1) and three YZ planes along the host artery, located 1mm, 5mm and 50mm distal from the toe of the anastomosis (i.e., Monitoring Planes 2–4).

In Fig 5, at Monitoring Plane 1, Models 2–4 show the maximum secondary velocity magnitude to be near the heel side and Model 4 shows significantly larger magnitude compared to the other models. Models 1–2 show symmetrical axial velocity distributions with the high-velocity crescent on the toe side. The out-of-plane helicity in Models 3–4 breaks flow symmetry. The maximum axial velocity magnitude is found in Models 2 and 4 because of having smaller graft cross-sectional area due to the ridge.

An important flow feature visible at Monitoring Plane 2 is the crescent-shaped peak secondary velocity region around the centre of the lumen towards the toe side. In the control model, two small symmetrical counter-rotating vortices are observed near the centre of the lumen, which are displaced and have become asymmetrical in the other models. The intensity of asymmetry is also increasing with the model number; this in turn can shift the maximum secondary velocity region to near-wall locations, which would result in an increase in the WSS at these regions. All four models show the presence of a separation region with a considerable size near the toe side.

At Monitoring Plane 3, two pairs of symmetrical counter-rotating vortices can be seen in the upper and lower recirculation regions in the control model. While the presence of four vortices for the control model are confirmed by plotting the λ_2_-criterion (see Section 3.3, below), the experimental data of Ha et al. [32,33] for a similar ETS configuration showed up to three vortices in the host artery. This discrepancy might be associated with the differences in the anastomosis angle (60° vs. 45°) and the maximum Reynolds number (1776 vs. 814) between the present model and the configurations tested in [32,33].

Adding the spiral ridge to the graft has resulted in an asymmetrical map for the secondary velocity magnitude. In Model 3, two main counter-rotating vortices can be identified in the upper half of the artery, with one being stronger than the other on. In Model 4, however, there are two similar vortices, one in each upper and lower half of the lumen.

The presence of large spatial velocity gradient from the lumen centre to the toe side is the most prominent flow feature in the axial velocity distributions at Monitoring Planes 2–3. However, including the non-planar helicity in Models 3–4 leads to smaller gradients and more uniform distributions, which can see as favourable conditions for the blood flow.

At Monitoring Plane 4, the weaker vortices seem to have dissipated in Models 1–3, while in Model 4, there is now one dominant vortex, with its core closer to the centre of the lumen. The maximum axial velocity magnitude at this section is found in Models 2 and 4, again as a result of having slightly higher bulk velocity due to the ridge.

At the reversed flow phase shown in Fig 6, very different distributions can be found at all four monitoring planes. At this phase, the maximum axial and secondary velocity magnitudes in the lumen have significantly been reduced and consequently, smaller spatial velocity gradients are found throughout the graft and the host vessel. In addition, compared to the maps shown in Fig 5, the peak secondary velocity regions in the reversed flow phase are generally larger in size and are shifted away from the wall towards the centre of the host artery. This has been further confirmed by the λ_2_-criterion, below. Another important finding from Fig 6 is that, introducing the spiral ridge produces negligible vortical structures in the host artery, while measurable secondary velocity magnitude is still visible in Models 3 and 4, due to their non-planar graft helicity.

### 3.3 λ_2_-Criterion

**Fig 7:**
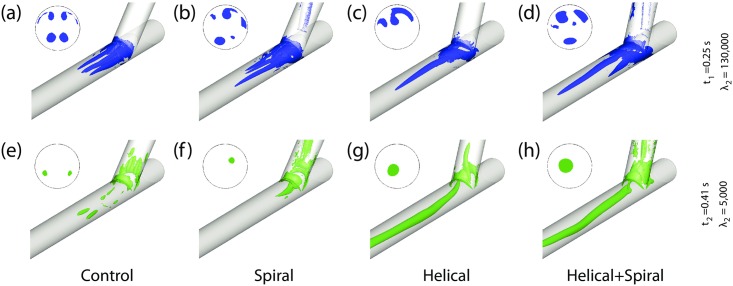
λ_2_ iso-surface of vortices within the present models at peak flow phase (*t*_1_ = 0.25s) and reversed flow phase (*t*_2_ = 0.41s), with λ_2_ iso-value thresholds of 130,000 and 5,000, respectively. Cross-sectional view at monitoring plane 3 (i.e., 5mm distal from the toe of the anastomosis) is also shown next to each model.

λ_2_-based vortex core detection [34] has been conducted to compare the size and location of vortices among the four different models. Fig 7 shows the λ_2_ iso-surface of vortices within the four models at time instances *t*_1_ and *t*_2_, with λ_2_ iso-value thresholds of 130,000 and 5,000, respectively.

At time *t*_1_, two pairs of symmetrical vortices are formed in Model 1 (Control), which lose their symmetry in Model 2 (Spiral), where the vortex near to the arterial floor has a higher elongation. In Model 3 (Helical), although there are two crescent-shape stretched vortical regions near the anastomosis, there is mainly one vortex, which is considerably elongated along the arterial axis. In Model 4 (Helical+Spiral), the effects of combining the out-of-plane helicity with an internal ridge can be observed clearly; there are three major vortical regions near the anastomosis, one of which is elongated considerably along the arterial axis (even longer than that in Model 3).

During the reversed flow phase, *t*_2_, a number of small vortical structures are formed at the anastomotic region of all models. However, in Models 3–4, a major vortex is visible at the centre of the host artery stretched along its axis. Therefore, consistent with the findings from Section 3.2, λ_2_ iso-surface of vortices within all the four models indicate the significance of the helical feature in the graft compared to the internal ridge in inducing and maintaining swirling flow even during the reversed flow phase.

### 3.4 Hemodynamic Parameters

There is currently extensive and increasing evidence, correlating the localisation of atherosclerosis, IH and Intimal Thickening (IT) to different local hemodynamic metrics [4,5,35]. Previous studies have shown that (i) localised distribution of low-WSS and high-Oscillatory Shear Index (OSI) strongly correlates with the focal locations of atheroma [36], and (ii) large spatial WSS Gradient (WSSG) contributes to the elevated wall permeability and atherosclerotic lesions [37], (iii) combination of high-shear stress and large exposure times may induce platelet activation [38–41], and (iv) stagnant and recirculation flow regions can cause platelet aggregation and thrombogenesis. Hence, in this study, the distributions of hemodynamic parameters, including Time-Averaged WSS (TAWSS), TAWSSG [42], OSI [36] and Relative Residence Time (RRT) [43,44] are calculated according to Eqs (3)–(6), respectively, and compared for all the four models.

$$TAWSS=\frac{1}{T}\int_{0}^{T}\left|{\vec{\tau }}_{W}\right|\;dt$$ (3)

$$TAWSSG=\frac{D_{G}}{\tau_{0}}\frac{1}{T}\int_{0}^{T}\sqrt{{\left(\frac{\partial \tau_{x}}{\partial x}\right)}^{2}+{\left(\frac{\partial \tau_{y}}{\partial y}\right)}^{2}+{\left(\frac{\partial \tau_{z}}{\partial z}\right)}^{2}}dt$$ (4)

$$OSI=\frac{1}{2}\left(1-\frac{\left|\int_{0}^{T}{\vec{\tau }}_{W}dt\right|}{\int_{0}^{T}\left|{\vec{\tau }}_{W}\right|\;dt}\right)$$ (5)

$$RRT=\frac{1}{\left(1-2\times OSI\right)\times TAWSS}=\frac{1}{\frac{1}{T}\left|\int_{0}^{T}{\vec{\tau }}_{W}dt\right|}$$ (6)

Here ${\vec{\tau }}_{W}$ is the WSS vector, *T* is the time period of the flow cycle, *D*_*G*_ is the graft diameter, and *τ*_0_ is the WSS for Poiseuille flow at the mean flow Reynolds number, which is calculated to be 0.7 Pa in this study.

**Fig 8:**
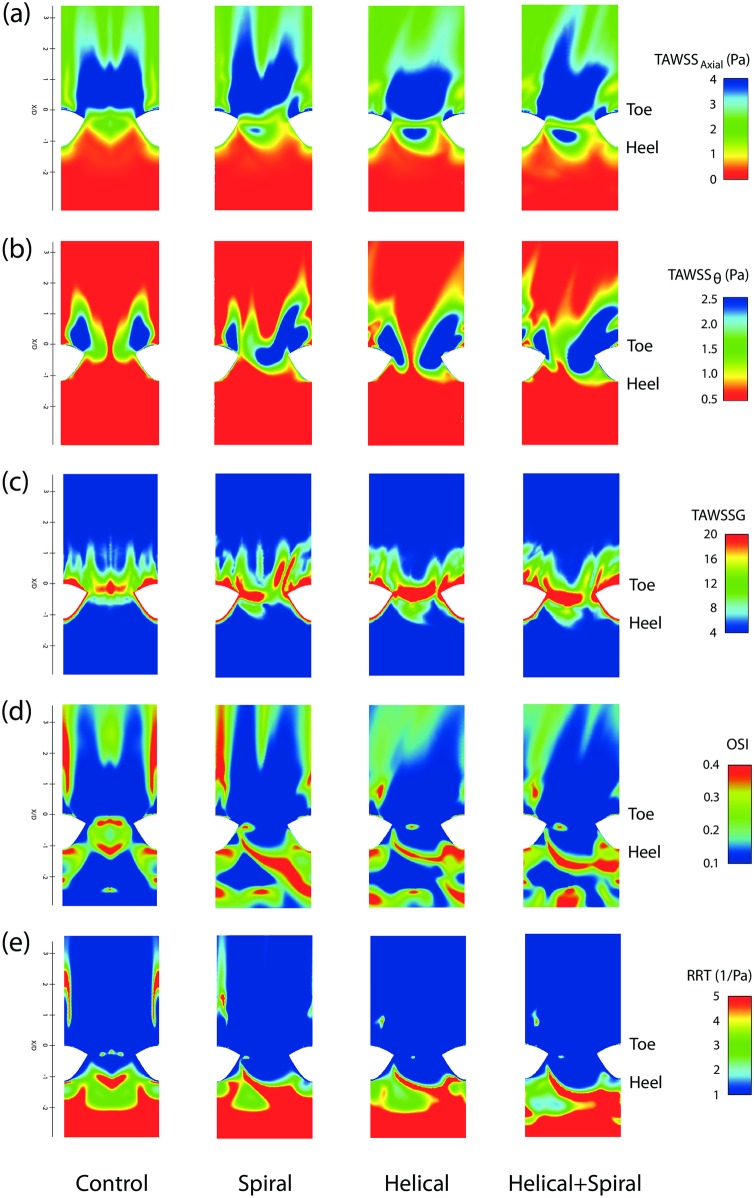
Distributions of different hemodynamic parameters viewed from the host arterial wall, opened ventrally and shown *en face*: (a) Axial Time-Averaged Wall Shear Stress (TAWSS_Axial_); (b) Radial Time-Averaged Wall Shear Stress (TAWSS_θ_); (c) Radial Time-Averaged Wall Shear Stress Gradient (TAWSSG); (d) Oscillatory Shear Index (OSI); and (e) Relative Residence Time (RRT). Note that the colour scale of the TAWSS maps in (a) and (b) are inverted for ease of comparison.

Fig 8 shows the distribution of the above metrics for all the four models displayed on an unfolded model of the host artery, which has been opened ventrally.

#### Time-Averaged Wall Shear Stress (TAWSS)

In the axial TAWSS distributions, shown in Fig 8a, there is a high-shear zone on the bed distal to the anastomosis in all four models, which is symmetrical for the Control model and asymmetrical in the other three models, with Model 4 having the highest degree of asymmetry. The stagnation line in all four models is represented by a small region of low-TAWSS across the anastomosis on the arterial bed. Adding the internal ridge and helicity features to the graft have resulted in higher TAWSS values in the region proximal to the stagnation point on the arterial bed (opposite the heel of the anastomosis); this high-shear region extends proximally towards the occluded section of the host artery as the secondary velocity magnitude in the graft is increased through combining the spiral ridge with helicity.

The distributions in Fig 8b show that all the four models have two main high-shear regions, which stretch distally from the toe on the wall of the host artery. Model 4 presents significant expansion of this high-TAWSS region in both distal and proximal directions.

#### Time-Averaged Wall Shear Stress Gradient (TAWSSG)

Non-dimensional TAWSSG distributions are shown in Fig 8c. As expected, elevated-TAWSSG regions are generally located near the separation and stagnation points, i.e., adjacent to the suture line and on the arterial bed across the toe. However, these regions are slightly less prominent in the Control model, compared to the other 3 models, particularly on the arterial bed, which is consistent with the observations from Fig 8a.

#### Oscillatory Shear Index (OSI)

Fig 8(d) compares the OSI distribution for all the four models, where it can be seen that the separation region distal to the toe and the stagnation point on the bed are associated with high OSI levels. The size and location of both the separation region and stagnation point are consistent with the observations from the TAWSS maps, discussed earlier.

In the Control model, there is an elevated-OSI region (split into 2 streaks on either side of the map), which is stretched distally from the toe on the arterial wall. In Model 2, the spatial extent of this region has partially been contracted, while in Models 3–4, they have been reduced significantly. Furthermore, there are distinct regions of elevated-OSI in the occluded segment of the host artery, which are generally associated with secondary flow oscillations; the spatial extent of the elevated-OSI regions increases as the spiral ridge and out-of-plane curvature are introduced to the graft. The elevated-OSI regions in Models 2–4 extend along a line, which is oblique to the vertical axis. This pattern represents the secondary flow oscillation due to rotation of the bulk flow within the anastomotic junction, which is more pronounced in Model 2, compared to Models 3–4. These findings are consistent with the numerical results of [13], despite the geometrical differences between the models.

#### Relative Residence Time (RRT)

While RRT has widely been used in the localisation of atherosclerotic diseases, it has rarely been calculated for bypass graft configurations. RRT would be a useful measure of the shear environment for correlative purposes that incorporates the level of the shear and its oscillatory character [43].

From the distributions in Fig 8e, elevated-RRT levels can be seen at the occluded segment of the host artery and in the separation regions distal to the toe of the anastomosis. The swirling flow induced by the helical graft in Models 3–4 has reduced the RRT values distal to the toe of the anastomosis, by eliminating the flow separation in this region (which was present in Model 1, and to a lesser extent in Model 2).

### 3.5 Flow Resistance

In order to assess the energy dissipation of the blood flow through the four different models, the resistance against the flow has been calculated under both steady-flow (with pressure boundary conditions: *P*_*inlet*_ = 320Pa, *P*_*outlet*_ = 0Pa) and pulsatile flow conditions. The results, presented in Table 2, showed that while the variation of resistance among different models was moderate, the graft with out-of-plane helicity (Model 3) causes the lowest increase in the graft resistance against the flow, as compared to Models 2 and 4.

**Table 2:** Resistance against the flow in the four geometric models.

	Model 1: Control	Model 2: Spiral	Model 3: Helical	Model 4: Helical+Spiral
**Steady Resistance (MPa∙s/m**^**3**^**)**	25.1	29.7	26	30.3
**Overall Oscillatory Resistance (MPa∙s/m**^**3**^**)**	61.53	74.75	65.04	78.07

The overall oscillatory resistance of the four models for the present pulsatile flow condition was calculated using Eq (7) [45]:

$$R=\frac{\int_{0}^{T}\left(P_{inlet}-P_{outlet}\right)Qdt}{\int_{0}^{T}Q^{2}dt}$$ (7)

where *Q* is the flow rate and *T* is the time period of the cardiac cycle.

The moderate variation of the resistance against the flow among different models justifies utilization of inlet flow condition (as opposed to inlet pressure boundary condition) in the present study.

### 3.6 Parametric Study of the Spiral Ridge

**Fig 9:**
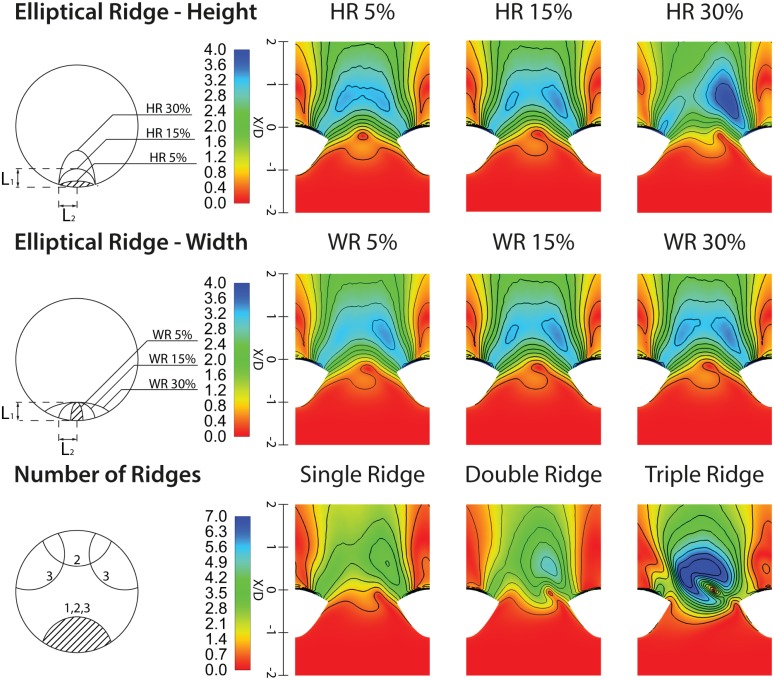
Distributions of normalised wall shear stress on an unfolded model of the host artery for different spiral ridge designs.

A series of steady-state flow simulations at a lower Reynolds number (*Re* = 570) was conducted for Model 2 (i.e., graft with internal ridge) to evaluate the effects of three different geometrical parameters, including the number of ridges as well as their heights and widths ratios. The details of the geometrical parameters tested here are given in Table 3. Fig 9 shows the normalised WSS distributions on an unfolded model of the host artery. WSS has been normalised with respect to the WSS of the Hagen-Poiseuille flow within a straight pipe of the same diameter (*D* = 6mm) and *Re* = 570. It can be seen that increasing the ridge Height Ratio (HR) has significant effects on increasing the magnitude and asymmetry of WSS distributions on the arterial bed, shifting the peak region away from bed centreline. The WSS distribution at the toe also increases with the ridge HR. However, changing the Width Ratio (WR) has minor effects.

**Table 3:** Summary of the parametric tests conducted on the end-to-side bypass graft with spiral ridge(s).

Cross-Sectional Designs *(Single ridge)*		Number of Ridges *(CSR = 15%)*
Ridge Height Ratio (HR) *(HR = L*_*1*_*/D & L*_*2*_ *= 0*.*9 mm)*	Ridge Width Ratio (WR) *(WR = L*_*2*_*/D & L*_*1*_ *= 0*.*9mm)*	
HR = 5% *(L*_*1*_ *= 0*.*3 mm)*	WR = 5% *(L*_*2*_ *= 0*.*3 mm)*	Single ridge
HR = 15% *(L*_*1*_ *= 0*.*9 mm)*	WR = 15% *(L*_*2*_ *= 0*.*9 mm)*	Double ridge
HR = 30% *(L*_*1*_ *= 1*.*8 mm)*	WR = 30% *(L*_*2*_ *= 1*.*8 mm)*	Triple ridge

It can also be seen that increasing the number of ridges has important effects on altering the WSS distributions on the bed of the host artery. In the triple-ridge case, a high-shear zone is visible on the arterial floor, distal to the stagnation point, where the maximum normalised WSS magnitude is approximately 85% and 50% higher than that in the single and double ridge cases, respectively. Higher number of ridges, however, would reduce the cross-sectional area and consequently increase the resistance against the flow. They may also result in an increase of pressure at the leading edge of the ridges, which can lead to deposition of cholesterol, calcium, cellular debris and fatty substances [46].

## 4 Discussion

The effectiveness of two novel graft designs, namely ‘SwirlGraft’ and ‘Spiral Flow Graft’ have been investigated in this study. While the main geometrical feature of the former has already been investigated numerically by a number of researchers [12–17], the effects of introducing a spiral ridge inside the graft and combining it with the non-planar helical design has not been studied before. The main findings from the present research will be discussed below in the contexts of the geometrical design and the clinical relevance of the hemodynamic parameters.

### 4.1 Geometrical Design Aspects

The present simulations indicated that combining the helical and spiral effects would result in the strongest secondary velocity magnitude in the host vessel. Furthermore, the non-planar helicity of the graft in Models 3 and 4 showed significant effects on breaking up the Dean vortices and producing predominantly single vortex. In fact, the numerical results of Lee et al. [17] have shown that generating a single vortex in the host artery is better compared to Dean-type vortices, since the latter would result in low wall shear regions associated with stagnation points. Besides, the platelet activation and adhesion tend to be significantly reduced under strong secondary flow conditions [47].

Comparing the distributions at peak- and reversed-flow phases reveals the role of helical and spiral grafts, which tend to generate a ‘sweeping’ motion, particularly in the host vessel. The sweeping motion of the vortices helps to expose the vessel wall to a higher range of WSS, which in turn plays a protective role against IH [48].

Effectiveness of out-of-plane helical structures over internal ridging is due to the fact that a helical graft induces the swirling flow effect onto the fluid near the graft lumen wall (i.e., within the boundary layers of the fluid where viscous forces are prevailing) and affects all around the perimeter of the graft lumen. Subsequently, the swirling effect is transferred all the way to the centre of the graft through friction between different layers of the fluid within the boundary layer. However, the spiral ridge only affects a small portion of the cross-sectional area at any given streamwise plane, and most of its effect is on the central regions of the lumen where viscous forces are not very strong. Moreover, lower resistance against the flow caused by the graft helicity (as compared to that caused by the spiral ridge), further boosts the superiority of the helical graft over the spiral graft.

When choosing different geometrical designs for cardiovascular prosthetics, it is important to sound a note of caution in relation to any attempt to find the ‘optimum’ graft design in the present bypass configuration solely by using conventional hemodynamic metrics. Substantial amount of work is required to construct an appropriate ‘cost function’ against which to perform shape optimisation. Moreover, for helical-based cardiovascular prostheses including the present configuration, additional metrics such as ‘helicity density’ [49] and ‘helicity-based bulk flow descriptors’ [50] can also be utilised to quantify the performance of each design (this is the subject of a future work for the present authors).

Finally, the paucity of *in vivo* data to support the existing hypotheses in the development of intimal hyperplasia (and atherosclerosis) in different bypass configurations is currently a major challenge [51], which also needs to be addressed in future studies.

### 4.2 Clinical Relevance of the Hemodynamic Metrics

The present computational results demonstrated that the spiral ridge is significantly less effective than the non-planar helicity of the graft, in producing vortical structures within the anastomosis and further downstream. In fact, in a very recent clinical investigation, Bechara [21] carried out a single-centre study on patients undergoing infrainguinal bypass using VFT’s spiral laminar flow graft against Propaten and found that the VFT’s graft had *not* led to higher patency rates in comparison to the conventional ePTFE grafts. This finding highlights the need for further investigation on the design and optimisation of the grafts with spiral ridges [46].

Investigation of the hemodynamic parameters in the present study revealed that TAWSS increases on the arterial bed and around the anastomosis by adding the non-planar helicity (while the effect of including the spiral ridge was minimal). There are indications that the blood monocytes are more likely to adhere to the endothelial layer at regions with low-TAWSS and high RRT [52]; hence, introducing the non-planar curvature to the graft may reduce the spatial extent of early wall lesion. Also, significant correlations have been reported between the preferred sites of IT and the regions of low-WSS [53,54]; as such the observed altered flow patterns may be perceived as a beneficial feature of the helical grafts which may positively impact the graft patency rate.

It is important to note that in addition to thrombosis, one should also consider the effects of mural platelet and fibrin deposits in the artery; the deposition of a mural layer of proteins and/or cells that can be the nidus for further cell and protein infiltration and atherosclerotic lesion progression, could subsequently result in graft failure. From the hemodynamic point of view, fibrinogen/fibrin is normally deposited at low shear rates and at areas exposed to eddies, flow separations and stasis (i.e. after stenosis and after areas of flow disturbance), thus, making RRT and WSS metrics even more relevant.

The present analyses also found that the elevated-OSI region on the host arterial wall (distal to the toe) reduces in size considerably in helical graft models (Models 3–4) and moderately in the spiral graft model. Hence, a low-TAWSS/high-OSI region in the control model is replaced by a high-TAWSS/low-OSI area in the helical graft models. This is another positive feature of introducing the non-planar helicity (and to a lesser extent the inclusion of the spiral ridge) to the bypass grafts, since the combination of high-TAWSS and low-OSI is believed to be contributing to IT and atherosclerosis development as well as increasing the risk of the aggregation of red blood cells [36,55,56].

In addition, the swirling flow induced by the helical graft in Models 3 and 4 reduces the particle residence time distal to the toe, by eliminating the flow separation in this region (which was present in Model 1 and to a lesser extent in Model 2). This could reduce the chance of platelet aggregation and thrombus formation [38], and consequently enhances the patency of the bypass graft.

## 5 Conclusions

The present study shows that inducing swirling flow into the bypass grafts would lead to positive flow features and more favourable distribution of hemodynamic parameters in the graft, anastomotic region, and the host artery, which in turn, could possibly enhance the patency and longevity of the bypass graft. To achieve swirling flow in the anastomosis, graft out-of-plane helicity was found to be significantly more effective than a single spiral ridge. It was also found that a combination of graft non-planar helicity and spiral ridge could further enhance the swirling effect in the flow. In light of the shortcomings of the graft with the spiral ridge in producing distinctive favourable hemodynamic conditions, a simple parametric study on the ridge geometrical features was conducted, which revealed that an elliptical ridge with large height ratio is preferred over a circular ridge and that the width ratio has minimal effects. It was also shown that the while multi-ridge designs reduce the effective cross-sectional area, they result in higher WSS magnitudes on the arterial bed. One of the limitations of the present study was the lack of detailed validation against *in vitro* data. Despite efforts in making qualitative comparison with geometrically-similar experimental data here, future studies should consider more rigorous validation to assess the accuracy of the numerical simulations. In addition, further studies are required in order to test a wider range of physical, geometrical and hemodynamic properties of both helical and spiral designs with a view to be utilised in different types of bypass grafts. This is the subject of a future work for the present authors.

## Supporting Information

S1 File Computer-Aided Design (CAD) file of the Control Model used in the present Study. (IGS)
